# A moderated mediation analysis of depression and age on the relationship between resilience and frailty among HIV-positive adults

**DOI:** 10.3389/fpubh.2023.1128309

**Published:** 2023-03-23

**Authors:** Lijun Meng, Dan Chen, Peiwu Hu, Meng Yao, Cui Zhou, Xingli Li

**Affiliations:** ^1^Department of Epidemiology and Health Statistics, Xiang Ya School of Public Health, Central South University, Changsha, Hunan, China; ^2^Wuhan Health Information Center, Wuhan, Hubei, China; ^3^Scientific Research Department, Xiangya Hospital of Central South University, Changsha, Hunan, China

**Keywords:** HIV/AIDS, adults, frailty, resilience, depression, age, moderated mediation

## Abstract

**Background:**

Given the continuing challenges frailty poses among people living with human immunodeficiency virus (HIV) (PLHIV), accumulating evidence suggests that frailty is linked to psychological factors. However, the mutual influences of resilience, depression, and frailty have not yet been clarified. This study aimed to identify the potential mechanistic pathway through which psychological factors mitigate frailty.

**Methods:**

Data were collected from June to August 2019 by trained investigators through face-to-face interviews with 375 HIV-positive Chinese adults. Each participant completed structured questionnaires to collect data in respect of their socio-demographic characteristics, and levels of frailty, depression, and resilience. These assessment measures included a self-designed questionnaire, the Tilburg Frailty Indicator (TFI), the 10-item Center for Epidemiological Studies Depression Scale (CES-D-10), and the 10-item Connor-Davidson Resilience Scale (CD-RISC-10). SPSS PROCESS macro was used to analyze the mediation and moderated mediation models.

**Results:**

The overall prevalence of frailty was 26.4%, and the prevalence of frailty among older and younger adults living with HIV was 22 and 31.4%, respectively. Mediation analysis showed that an association between resilience and frailty was mediated by depression, whereas resilience did not mediate the relationship between depression and frailty. Compared to physical frailty, depression was a stronger mediator of resilience to psychological frailty. We further found that age moderated the indirect effect of resilience on psychological frailty, with resilience being a stronger negative predictor of depression and depression being a stronger positive predictor of psychological frailty for older PLHIV than for younger PLHIV.

**Conclusion:**

Lower levels of resilience and greater levels of depression may be significant risk factors for frailty among PLHIV. Levels of resilience influenced frailty directly and frailty was indirectly affected by depression. Therefore, it is recommended that PLHIV, especially older patients, should be encouraged to establish positive psychological coping strategies to slow the progression of frailty.

## 1. Introduction

With the extensive use of highly effective antiretroviral therapy, the human immunodeficiency virus (HIV) has gradually been transformed from a lethal infection to a manageable chronic condition, allowing a growing number of people living with HIV (PLHIV) to survive longer with increased quality of life (1, 2). A Dutch cohort study further estimated that by 2030, the median age of HIV- infected patients would increase by 12.7 years compared to the age in 2010, with the proportion of patients aged 50 years or older increasing by 73% (3). These developments have required a greater focus on reducing PLHIV’s vulnerability to the adverse effects associated with aging.

Frailty is a common geriatric syndrome that results from the interactions of an individual’s physical, psychological, and social environment, which can lead to a series of adverse consequences (4). Previous studies found that HIV infection may accelerate the onset of the detrimental consequences of aging, leaving PLHIV to face the challenge of age-related diseases at an earlier age than their uninfected peers (5, 6). Compared with individuals who are not infected, frailty occurs approximately 10–15 years earlier, and has a higher prevalence in PLHIV, ranging from 5 to 28.6% (7, 8). Although many corresponding indicators of frailty have been identified, there is little known about the psychosocial factors related to frailty among PLHIV.

Resilience is a psychosocial factor that refers to an individual’s ability to recover from negative experiences and adapt well to adversity (9). It is becoming increasingly important as a protective factor against negative psychological health outcomes that may enable PLHIV to overcome the negative effects of the challenges facing this specific subsection of the population (10, 11). Studies on non-HIV-infected populations have demonstrated that resilience is inversely correlated with frailty, implying that people with low levels of resilience may experience higher rates of frailty (8, 12). However, the mechanisms underlying the effects of resilience on frailty remain unclear. As a result of this we developed Hypothesis 1 (*H1*) as follows: Resilience is negatively associated with frailty.

Depression, a common mental disorder in PLHIV, is another focus area of research on risk factors of frailty (13). There is mounting evidence of a high prevalence of depression among PLHIV due to the number of chronic HIV-related problems experienced by PLHIV, such as social stigma or inadequate social support (14), with estimates that up to 80% of patients are affected. With this precept, hypothesis 2 (*H2*) was developed in the present study: Depression is positively predictive of frailty. Extensive research on non-HIV infected populations has presented results that suggest that severe depressive symptoms are associated with lower levels of resilience and greater levels of frailty (13, 15, 16). Resilience is a factor known to be associated with positive health outcomes, thus hypothesis 3 (*H3*) was proposed: Resilience plays a mediating role in the relationship between the relationship between depression and frailty. Also, considering the relationship between low resilience and frailty, other potential factors may be involved in the progression from low levels of resilience to severe frailty. Prior studies have examined the mediating role of depression in other relationships (17, 18), yet few studies have explored the underlying mediating mechanisms between resilience and frailty. In light of this, Hypothesis 4 (*H4*) was developed: Depression plays a mediating role in the relationship between resilience and frailty.

Moreover, with the increase in life expectancy and the extension of the period of aging among PLHIV, age-related differences in various health indicators have become more pronounced. On the one hand, the incidence of frailty increases with age, and the risk of occurrence is particularly high in PLHIV aged 50 years or older (7, 19). However, a large body of research has shown that there are some differences in the physical and mental health of PLHIV across age groups, with older adults generally facing additional psychological challenges when compared to their uninfected counterparts (20–22). Therefore, the relationship between resilience and frailty or depression and frailty has different mediating effects and it would seem that these effects are dependent on the age of the individual. Consequently, Hypothesis 5 (*H5*) was developed as follows: Age is a moderator of the mediating effect of depression on the relationship between resilience and frailty or resilience on the relationship between depression and frailty.

**Figure 1:**
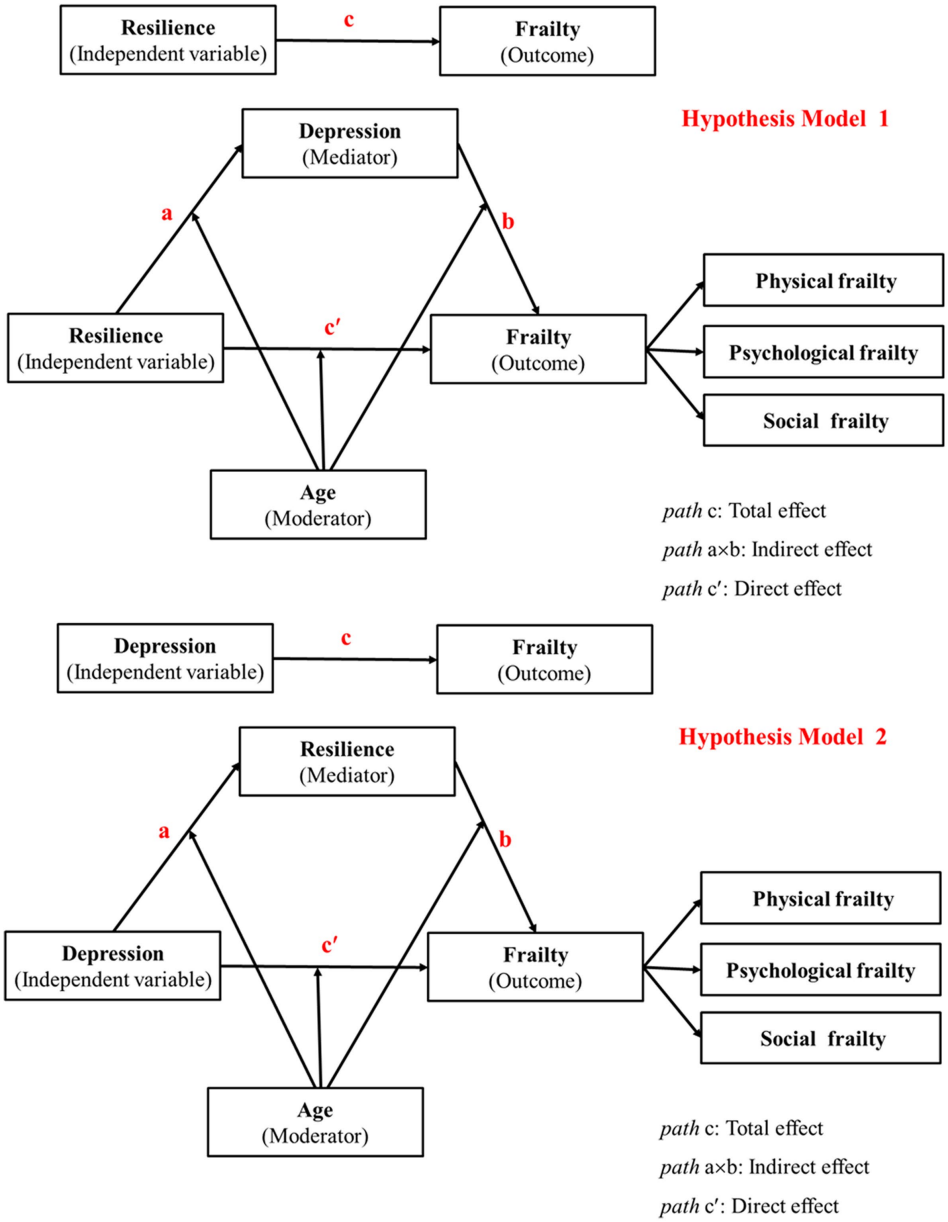
Hypothesis models of age as a moderator of the relationship between resilience, depression and frailty.

To investigate the above five hypotheses and determine the potential mediating mechanism affecting frailty in a sample of HIV-positive Chinese adults, two moderated mediation models were constructed (Figure 1). To the best of our knowledge, there are no previous studies that have been conducted on the effect of psychological factors on frailty in this particular population, and even less is known about the mediating role of the relationship between the variables. We also intended to test the theory that age moderates the mediating relationship associated with frailty.

## 2. Materials and methods

### 2.1. Participants and procedure

We selected the First Hospital of Changsha City as the study site since it is the municipal designated hospital for HIV and acquired immune deficiency syndrome (AIDS) treatment in the provincial capital city of Hunan Province. As such it attracts patients with characteristics different from those of most of the surrounding prefecture-level cities. We performed a cross-sectional study of patients receiving in-or out-patient treatment at this hospital for the period June to August 2019. Data in respect of the patients clinical characteristics were obtained from the Chinese AIDS registration system. A pre-survey was conducted before the official start of the study, and the contact details of the participants were obtained. After the participants completed the survey all questionnaires were checked to ensure that they had been completed correctly and in full, and 22 invalid questionnaires had to be excluded.

In the present study, the sample size was estimated based on the formula for the cross-sectional survey being: *n*= $\frac{Z_{\alpha /2}^{2}\times p\left(1-p\right)}{\delta^{2}}$ , where $Z_{\alpha /2}$ was the statistic of a significance test, *p* was the expected prevalence rate, and δ was the margin of error. Considering the previous literature on the prevalence of frailty in PLHIV of 40%, α=0.05,δ=0.08, a sample size of at least 150 individuals was calculated. Considering the validity of the questionnaire and the possibility of incomplete or invalid responses, it was decided that the sample size should be increased appropriately. The participants had to meet the following inclusion criteria: (i) adults (aged ≥18 years) who were not pregnant or lactating, (ii) diagnosed with HIV infection or AIDS, and (iii) understood the aim of the study and were willing and able to complete the questionnaire. This study was designed in accordance with the principles of the Declaration of Helsinki and was approved by the Ethical Review Committee of the Xiangya School of Public Health, Central South University (XYGW-2019-20). Informed consent was obtained from the participants before the study began and all data were analyzed after ensuring the participant’s details were to be kept confidential (e.g., name and contact details).

### 2.2. Measures

#### 2.2.1. Socio-demographic characteristics

We conducted face-to-face interviews with a self-designed questionnaire to obtain demographic information and assess HIV-related factors, including age (18–49 years old, ≥50 years old), sex (men, women), ethnicity (Han ethnicity, minority), place of residence (rural, urban), employment status (employed, unemployed), level of education (primary school or less, junior school or above), marital status (married/cohabiting, divorced/separated, other), living status (lives alone, lives with others), monthly income (up to ¥3,000, over ¥3,000), period of infection (<5 years, 5–9 years, ≥10 years), showing clinical symptoms/signs (yes, no) and route of transmission (homosexual transmission, heterosexual transmission, other/unknown). Of these, clinical symptoms/signs refer to the presence of persistent fever, ulcerated rash, thrush, swollen lymph nodes, and other HIV-related symptoms or signs.

#### 2.2.2. Frailty

The Tilburg Frailty Indicator (TFI) is a 15-item questionnaire, including three dimensions of frailty, namely physical frailty (8 items), psychological frailty (4 items) and social frailty (3 items). The questionnaire was designed to quantitatively assess frailty status, with a total TFI score of ≥5 rendering an individual frailty positive (23). Sample items from the scale included “Have you experienced a sudden and significant weight loss?” “Did you feel tired often?” “Did you receive enough help from others?” The responses to all items were scored and added together to obtain a total frailty score ranging from 0 to 15, with higher scores indicating a more severe level of frailty. Previous studies on the Chinese population have demonstrated that the TFI has good cultural adaptability, reliability and validity (24). The Cronbach’s α coefficient was 0.677 for the total scale in the present study.

#### 2.2.3. Depression

The Center for Epidemiological Studies Depression Scale (CES-D), which consists of 10 items taken from the original 30-item CES-D Scale (25), was used to evaluate the mental health of the participants during the preceding week. The participants’ responses were recorded on a 4-point Likert scale (almost never = 0, sometimes = 1, often = 2, most of the time = 3), and the items “hopeful for the future” and “I am very happy” were reverse scoring items. The possible total score ranged from 0 to 30, with higher scores indicating greater depressive symptoms. The Cronbach’s α coefficient of the scale in this sample was 0.814.

#### 2.2.4. Resilience

The Connor-Davidson Resilience Scale-10 (CD-RISC-10) (26) was utilized to measure resilience, which refers to the process and ability of individuals to successfully adapt and recover from adversity or difficult life experiences. Each item was scored on a 5-point Likert scale (never = 1, rarely = 2, sometimes = 3, usually = 4, always = 5), and the total score ranged from 10 to 50, with higher scores reflecting better resilience. The Cronbach’s α coefficient for the present study was 0.879.

### 2.3. Data analysis

Data were analyzed using IBM SPSS Statistics (version 25.0; SPSS Inc., Chicago, United States). Descriptive statistics involving the demographic characteristics of the participants and correlation analysis of the key variables were examined. The mediation and moderated mediation models were tested using the PROCESS macro program for SPSS (*Model 4 and Model 59*) developed by Hayes.^1^ The bias-corrected non-parametric percentile bootstrap method was used for parameter estimation. In total 5,000 random samples were used to generate bootstrap confidence intervals to verify whether the indirect effects were significant. If the 95% confidence interval (*CI*) did not include zero, the coefficients and indirect effects were to be considered significant.

All *p* values were two-tailed, with the significance level set at 0.05. In addition, employment status, level of education, monthly income, and presence of clinical symptoms/signs were controlled for as covariates in all models, and the study variables were standardized.

## 3. Results

### 3.1. Demographic characteristics of participants

A total of 375 PLHIV were interviewed. Their ages ranged from 20 to 79 years, with an average age of 48.26 years. The majority of participants were men (71.2%), of Han ethnicity (95.7%), married (59.2%), and living with others (77.1%). Regarding education level, 81.1% were junior school graduates or higher. In this study, most infections were transmitted sexually. In addition, the prevalence of frailty was higher among participants who were older, more educated, unemployed, had a lower income, and had clinical symptoms or signs (Table 1).

**Table 1:** Different demographic characteristics and the distribution of frailty (*n* = 375).

Characteristic	*N* (%)	Frailty (*n*,%)		*χ^2^*	*p*
		Yes (*n* = 99)	No (*n* = 276)		
Age (years old)					
18 ~ 49	200 (53.3)	44 (44.4)	156 (56.5)	4.270	0.039^*^
≥50	175 (46.7)	55 (55.6)	120 (43.5)		
Sex					
Men	267 (71.2)	70 (70.7)	197 (71.4)	0.016	0.900
Women	108 (28.8)	29 (29.3)	79 (28.6)		
Ethnicity					
Han ethnicity	359 (95.7)	93 (93.9)	266 (96.4)	1.060	0.303
Minority	16 (4.3)	6 (6.1)	10 (3.6)		
Residence					
Urban	177 (47.2)	51 (51.5)	126 (45.7)	1.005	0.316
Rural	198 (52.8)	48 (48.5)	150 (54.3)		
Employment status					
Employed	220 (58.7)	47 (47.5)	173 (62.7)	6.948	0.008^**^
Unemployed	155 (41.3)	52 (52.6)	103 (37.3)		
Level of education					
Primary school or less	71 (18.9)	26 (26.3)	45 (16.3)	4.708	0.030^*^
Junior school or above	304 (81.1)	73 (73.7)	231 (83.7)		
Marital status					
Married/Cohabiting	222 (59.2)	58 (58.6)	164 (59.4)	0.543	0.762
Divorced/Separated	67 (17.9)	16 (16.2)	51 (18.5)		
Other	86 (22.9)	25 (25.3)	61 (22.1)		
Living status					
Lives alone	86 (22.9)	29 (29.3)	57 (20.7)	3.078	0.079
Lives with others	289 (77.1)	70 (70.7)	219 (79.3)		
Monthly income					
Up to ¥3,000	178 (47.5)	58 (58.6)	120 (43.5)	6.669	0.010^*^
Over ¥3,000	197 (52.5)	41 (41.4)	156 (56.5)		
Length of infection (years)					
<5	104 (27.7)	33 (33.3)	71 (25.7)	4.573	0.102
5 ~ 9	145 (38.7)	41 (41.4)	104 (37.7)		
≥10	126 (33.6)	25 (25.3)	101 (36.6)		
Clinical symptoms/signs					
Yes	205 (54.7)	80 (80.8)	125 (45.3)	37.092	<0.001^**^
No	170 (45.3)	19 (19.2)	151 (54.7)		
Route of infection					
Homosexual transmission	98 (26.1)	21 (21.2)	77 (27.9)	2.048	0.359
Heterosexual transmission	233 (62.1)	64 (64.6)	169 (61.2)		
Others/Unknown	44 (11.7)	14 (14.1)	30 (10.9)		

^*^p < 0.05, ^**^p < 0.001. Comparison between groups using chi-squared test. Bold *p* values indicate statistical significance.

### 3.2. Correlation between resilience, depression, and frailty

The key variables were not normally distributed according to the results of the Kolmogorov–Smirnov test; thus, the Spearman rank correlation test was used to explore the relationships between the variables. The results of the correlation analysis showed that depression was negatively correlated with resilience (*r_s_* = −0.658, *p* < 0.01), whereas it was positively correlated with total frailty (*r_s_* = 0.593, *p* < 0.01). The dimension of social frailty was associated with neither depression nor resilience (*p* > 0.05). In addition, higher levels of resilience were associated with lower levels of frailty (*r_s_* = −0.47, *p* < 0.01), and older adults reported experiencing increased frailty (*Z* = −3.176, *p* < 0.01; Table 2).

**Table 2:** Age difference and correlation among resilience, depression and frailty.

Variable	Younger PLHIV (*n* = 200)	Older PLHIV (*n* = 175)	1	2	3	4	5
	*M* (*IQR*)	*M* (*IQR*)					
1. Total frailty scores	3.00 (3.00)	3.5 (3.5)^**^	1.00				
2. Physical frailty scores	1.00 (2.00)	2.00 (3.00)	0.832^**^	1.00			
3. Psychological frailty scores	1.00 (1.00)	1.00 (1.00)	0.649^**^	0.376**	1.00		
4. Social frailty scores	1.00 (1.00)	1.00 (1.00)	0.316^**^	−0.052	−0.028	1.00	
5. Resilience scores	40.00 (11.00)	39.00 (10.00)	−0.470^**^	−0.387^**^	−0.493^**^	0.007	1.00
6. Depression scores	7.00 (6.00)	6.00 (8.00)	0.593^**^	0.454^**^	0.682^**^	0.010	−0.658^**^

M, median; IQR, interquartile range. ^**^p < 0.01. Comparison between groups using Mann–Whitney *U*-test.

### 3.3. Testing the mediating role of depression and resilience

Results of the mediating effect between resilience and frailty through depression were shown in Table 3. The total effects of different pathways were significant except for the social frailty dimension, indicating an association between resilience and frailty, in particular that resilience negatively predicted frailty. In addition, the indirect effects between resilience and total frailty or physical frailty or psychological frailty through depression were all significant, supporting the mediating role of depression in the relationship between resilience and frailty was present and valid. The proportion of the mediating effects of depression in total frailty, physical frailty and psychological frailty were 82.2, 56.4, and 88.2%, respectively.

**Table 3:** Results of mediating effect for depression between resilience and frailty.

Paths	Bootstrap effects					
	Total effect	LLCI	ULCI	Indirect effect	LLCI	ULCI
Resilience → Depression → Total frailty	−0.455	**−0.542**	**−0.367**	−0.374	**−0.461**	**−0.290**
Resilience → Depression → Physical frailty	−0.477	**−0.618**	**−0.336**	−0.269	**−0.364**	**−0.184**
Resilience → Depression → Psychological frailty	−0.491	**−0.565**	**−0.418**	−0.433	**−0.510**	**−0.361**
Resilience → Depression → Social frailty	0.004	−0.075	0.084	−0.013	−0.100	0.070

LLCI, Lower Limit 95% Confidence Interval; ULCI, Upper Limit 95% Confidence Interval. Bold numbers in the table indicated statistically significant at 0.05, which bootstrap interval did not include 0.

Results of the mediating effect between depression and frailty through resilience were shown in Table 4. The total effect of depression on total frailty, physical frailty, and psychological frailty was found to be significant, showing that depression was positively associated with frailty. Contrary to our expectations, no statistically significant indirect effects of the different pathways on frailty were observed, revealing that resilience did not play a mediating role in the relationship between depression and frailty.

**Table 4:** Results of mediating effect for resilience between depression and frailty.

Paths	Bootstrap effects					
	Total effect	LLCI	ULCI	Indirect effect	LLCI	ULCI
Depression → Resilience → Total frailty	0.586	**0.510**	**0.662**	0.054	−0.204	0.127
Depression → Resilience → Physical frailty	0.627	**0.497**	**0.758**	0.046	−0.079	0.167
Depression → Resilience → Psychological frailty	0.608	**0.548**	**0.669**	0.081	−0.018	0.142
Depression → Resilience → Social frailty	0.007	−0.070	0.010	−0.012	−0.094	0.064

LLCI, Lower Limit 95% Confidence Interval; ULCI, Upper Limit 95% Confidence Interval. Bold numbers in the table indicated statistically significant at 0.05, which bootstrap interval did not include 0.

### 3.4. Testing the moderating effect of age

Moderated mediation analysis was performed in a valid mediating relationship. For total frailty and physical frailty, age only moderated *Path a*, more specifically, age moderated the predictive ability of resilience for depression. However, age did not play a moderating role in *Path b* and direct pathway (*Path c′*). In terms of psychological frailty, age moderated both the first and the second half of the indirect effect of the relationship between resilience and frailty mediated by depression (Table 5). As shown in Table 6, there was convincing evidence that the indirect effect was stronger for older PLHIV than for younger PLHIV, in which the 95% bootstrap *CI* did not include 0. Compared to younger PLHIV, resilience was a stronger negative predictor of depression and depression was a greater positive predictor of psychological frailty in older PLHIV.

**Table 5:** Results of age as a moderator on the relation between resilience on frailty *via* depression.

Outcome, predictor	Indicator		*β*	*SE*	*t*	LLCI	ULCI
	*R^2^*	*F*					
*Depression*							
Resilience × Age	0.510	54.518^**^	−0.196	0.074	−2.648^**^	**−0.342**	**−0.050**
*Total frailty*							
Depression × Age	0.501	40.791^**^	−0.059	0.105	−1.580	−0.372	0.041
Resilience × Age			−0.166	0.107	−0.232	−0.411	0.009
*Physical frailty*							
Depression × Age	0.356	22.436^**^	−0.058	0.183	−0.319	−0.418	0.301
Resilience × Age			−0.332	0.181	−1.835	−0.688	0.024
*Psychological frailty*							
Depression × Age	0.566	59.410^**^	−0.213	0.085	−2.511^*^	**−0.380**	**−0.046**
Resilience × Age			−0.162	0.084	−1.936	−0.327	0.003

^*^p < 0.05, ^**^p < 0.001. LLCI, Lower Limit 95% Confidence Interval; ULCI, Upper Limit 95% Confidence Interval. Bold numbers in the table indicated statistically significant at 0.05, which bootstrap interval did not include 0.

**Table 6:** Results of the moderating effect of age in the indirect effects of resilience on psychological frailty.

Groups	Resilience → Depression			Depression → Psychological frailty		
	Effect	LLCI	ULCI	Effect	LLCI	ULCI
Younger PLHIV	−0.601	**−0.694**	**−0.508**	0.437	**0.522**	**0.779**
Older PLHIV	−0.767	**−0.880**	**−0.655**	0.650	**0.329**	**0.545**

LLCI, Lower Limit 95% Confidence Interval; ULCI, Upper Limit 95% Confidence Interval. Bold numbers in the table indicated statistically significant at 0.05, which bootstrap interval did not include 0.

## 4. Discussion

In this study, a moderated mediation model of frailty involving resilience, depression, and age was established in HIV-positive adults. According to our results, the major findings are as follows: (i) frailty was not only highly prevalent among older PLHIV, but also among younger PLHIV; (ii) resilience could predict frailty directly or indirectly, affecting frailty *via* depression; (iii) resilience does not play a mediating role in the relationship between depression and frailty; and (iv) age moderated the indirect effect of resilience on psychological frailty, with the effect of resilience on depression or the effect of depression on psychological frailty being more strongly associated with older PLHIV than with younger PLHIV. Our findings provide the first epidemiological evidence of the mediating role of depression in the relationship between resilience and frailty in HIV-positive adults, implying that the onset of frailty can be mitigated to a certain extent through the development of greater resilience and reduction of depressive symptoms.

The occurrence of frailty among PLHIV not only depends on aging, psychology and social environment but also on the adverse consequences of HIV infection experienced by PLHIV. Mitochondrial dysfunction induced by antiretroviral drug therapy in PLHIV may be associated with increased levels of frailty, causing frailty to occur not only in older PLHIV (27). In addition, the existing literature provides evidence that immune system dysfunction and persistent chronic inflammation caused by HIV infection accelerates the onset and degenerative process of frailty (28, 29). In the present study, frailty was more common in older PLHIV than in younger PLHIV, which is consistent with previous studies (30, 31). In addition, the overall prevalence of frailty among our participants was 26.4%, which is higher than the results of a meta-analysis of older individuals in the Chinese community (which ranged from 5.9 to 17.4%) (32). As frailty can be improved by implementing a wide range of interventions, we submit that clinicians should focus their attention on attempting to prevent and slow down the onset and process of frailty.

The previous theoretical framework model considered psychological functioning to have a greater impact on frailty than physical function (33), therefore we chose to examine the effects of resilience and depression on frailty in this study. We found a strong negative association between frailty and resilience as well as a strong positive association with depression, with the ultimate results supporting and confirming *H1 and H2*. This finding validates existing evidence that resilience has been reported to be a protective factor against the low health-related quality of life problems such as disability and chronic disease (34, 35), This suggests that high levels of resilience may be useful in improving the well-being and quality of life of PLHIV who experience the onset of frailty. Consistent with the results of previous studies, depression was found to be a risk factor for frailty (36), indicating that depressed PLHIV are more likely to develop frailty.

Furthermore, this study explored the mediating role of depression in the relationship between resilience and frailty among PLHIV. The results confirmed the validity of *H4* and suggested that resilience not only affected frailty directly but also indirectly *via* depression. It is well known that PLHIV suffer many negative physical, psychological, and financial consequences, which may lead to low levels of resilience and eventually depression (37). The evidence to date supports the notion that depression and frailty are distinct structures with moderate overlap. A review of recent clinical and preclinical evidence has emphasized that depression is a risk factor for frailty, which may be explained by several mechanisms. Depressive disorder, a comorbid condition in PLHIV, is associated with impaired motivation and reduced treatment adherence (38). It has also been suggested that individuals with depression have high levels of inflammatory cytokines, which may stimulate the brain and lead to changes in nervous system function (39, 40). Elevated levels of inflammatory cytokines are strongly associated with the onset of neurological dysfunction in the brain (41), and similar biological mechanisms provide a plausible explanation for the strong correlation between depression and frailty.

Unfortunately, resilience had no indirect effect on the relationship between depression and frailty, implicating that resilience did not play a mediating role. This was unexpected, as a mediating role for resilience was found in previous studies (42). Clearly, a greater impact of depression on frailty compared to the impact of resilience on frailty contributed to the fact that the effect of depression on frailty was not mediated by psychological resilience in this study. Also, it is difficult to compare our sample to the geriatric population and to assess resilience and frailty with different measurements compared to other similar studies.

We further found that age moderated both the first and second half pathways of the indirect effect of resilience on psychological frailty, whereas only the first half of the pathway on physical frailty was moderated. The moderating effect on the relationship between resilience and depression was more pronounced in older PLHIV than in younger patients, indicating that resilience is a stronger negative predictor of depression in older PLHIV. Resilience may be associated with improvements in physical and psychological health and it is likely to increase with age. Earlier studies on the general population confirmed that older people have higher levels of resilience than younger people (34). Older people with high levels of resilience usually have more life experience and are able to cope better with adversity and stress than younger people. In addition, depression was more predictive of psychological frailty in older PLHIV compared to younger PLHIV, which is associated with older PLHIV experiencing the shock of aging, disease distress and HIV infection simultaneously (43). Unexpectedly, no association was evident between the direct and the second half of the indirect pathway of resilience to physical frailty across age subgroups. This suggests a stronger association between physical frailty and aging than the association with psychological function (44).

## 5. Limitations

This study has several limitations. First, the sample was selected only from the patients who attend the First Hospital of Changsha City, which is not representative of all PLHIV. Second, the data were obtained from participants’ subjective reports and may be susceptible to participant bias. Third, this was a cross-sectional research design based on a questionnaire, and the results may not be able to fully determine the causal relationships between the variables. Future longitudinal studies with larger sample sizes are needed to explore the underlying mechanisms of frailty in PLHIV in greater detail. In addition, the complex etiology of frailty requires multidisciplinary intervention and treatment. There may also be other related factors that mediate or moderate the relationship between resilience and frailty, which necessitates further investigation.

Despite these limitations, this study revealed an association between resilience, depression, and frailty in PLHIV. In addition, it also identified a potential pathway for the occurrence of frailty in PLHIV that could provide new insights and suggestions for reducing the incidence of frailty by encouraging positive coping mechanisms in the face of negative events and increasing levels of resilience to buffer against depression.

## 6. Conclusion

The present study revealed that the issue of frailty in PLHIV should not be trivialized and that the current status of frailty in both older and younger PLHIV is not optimistic. Moreover, we found an association between an early onset of frailty and low levels of resilience, which was mediated by depression. Thus, in addition to focusing on the impact of physical functioning on frailty, it is critical for healthcare staff to take appropriate measures to assist patients develop effective psychological coping strategies to improve mental resilience, especially in older patients. In implementing such interventions, it would be beneficial to promote the maintenance of psychological well-being in PLHIV to reduce depression and ultimately to attempt to mitigate the consequences of frailty to a certain extent.

## Data availability statement

The original contributions presented in the study are included in the article/supplementary material, further inquiries can be directed to the corresponding author.

## Ethics statement

The studies involving human participants were reviewed and approved by the Ethical Review Committee of Xiangya School of Public Health, Central South University (XYGW-2019-20). The patients/participants provided their written informed consent to participate in this study.

## Author contributions

LM: methodology, formal analysis, roles and writing-original draft, and writing-review and editing. DC: investigation, data curation, and methodology. PH: supervision, validation, visualization, and resources. MY: investigation and formal analysis. CZ: validation and visualization. XL: funding acquisition, project administration, and writing-review and editing. All authors contributed to the article and approved the submitted version.

## Funding

This research was supported by the Natural Science Foundation of Hunan Province (grant no. 2022JJ30779).

## Conflict of interest

The authors declare that the research was conducted in the absence of any commercial or financial relationships that could be construed as a potential conflict of interest.

## Publisher’s note

All claims expressed in this article are solely those of the authors and do not necessarily represent those of their affiliated organizations, or those of the publisher, the editors and the reviewers. Any product that may be evaluated in this article, or claim that may be made by its manufacturer, is not guaranteed or endorsed by the publisher.

## Footnotes

^1^ http://www.processmacro.org
